# Metformin Is Associated with a Lower Incidence of Benign Brain Tumors: A Retrospective Cohort Study in Patients with Type 2 Diabetes Mellitus

**DOI:** 10.3390/biom11101405

**Published:** 2021-09-25

**Authors:** Chin-Hsiao Tseng

**Affiliations:** 1 Department of Internal Medicine, National Taiwan University College of Medicine, Taipei 10051, Taiwan; ccktsh@ms6.hinet.net; Tel./Fax: +886-2-2388-3578; 2 Division of Endocrinology and Metabolism, Department of Internal Medicine, National Taiwan University Hospital, Taipei 10002, Taiwan; 3 National Institute of Environmental Health Sciences, Zhunan 35053, Taiwan

**Keywords:** benign brain tumors, cerebral meningioma, diabetes mellitus, metformin, Taiwan

## Abstract

**Background**: The risk of benign brain tumors (BBT) associated with metformin use has not received much attention. Therefore, a retrospective cohort study was designed to investigate such an association in patients with type 2 diabetes mellitus (T2DM). **Methods**: We used the database of Taiwan’s National Health Insurance to enroll 152,176 ever users and 16,120 never users of metformin for the follow-up of incidence of BBT and a more specific outcome of cerebral meningioma. The patients were newly diagnosed with T2DM between 1999 and 2005; and they were followed up from 1 January 2006 until 31 December 2011. Hazard ratios were estimated by Cox regression incorporated with the inverse probability of treatment weighting using propensity score. **Results**: During follow-up, 111 never users and 557 ever users were diagnosed with BBT. For BBT, the respective incidence rates for never users and ever users were 153.95 per 100,000 person-years and 77.61 per 100,000 person-years. While ever users were compared to never users, the hazard ratio was 0.502 (95% confidence interval: 0.409–0.615). A dose-response pattern was seen when ever users were categorized into tertiles of cumulative duration of metformin therapy (cutoffs: <27.10 months, 27.10–58.27 months and >58.27 months) with respective hazard ratios of 0.910 (0.728–1.138), 0.475 (0.375–0.602) and 0.243 (0.187–0.315). For cerebral meningioma, the overall hazard ratio was 0.506 (0.317–0.808); and the hazard ratios comparing the respective tertiles to never users were 0.895 (0.531–1.508), 0.585 (0.346–0.988) and 0.196 (0.104–0.369). **Conclusions:** A reduced risk of BBT and cerebral meningioma is observed in metformin users in patients with T2DM.

## 1. Introduction

Meningioma is the most common benign brain tumor (BBT) (53.2%) [1] and may be associated with metabolic syndromes, obesity, diabetes mellitus, hypertension and lack of physical activity [2,3,4,5]. There is probably no association with smoking, alcohol drinking or dietary factors [6,7]. On the other hand, the roles of hormone use, ionizing radiation, cell phone use and some occupational exposures are not clear [8].

Metformin is an old oral antidiabetic drug that has been used since the 1940s [9]. It inhibits hepatic glucose output from gluconeogenesis and stimulates glucose uptake into skeletal muscle. The mechanism of these metabolic effects of metformin is mediated by the inhibition of mitochondrial respiratory chain complex 1, which in turn activates the 5′ adenosine monophosphate-activated protein kinase (AMPK) [9]. Because of the potential risk of fatal lactic acidosis, its use was banned in the USA and Australia until 1995 [9]. Its use has gained momentum after 1998 when a reduced risk of cardiovascular events in obese/overweight patients with type 2 diabetes mellitus (T2DM) was demonstrated by the United Kingdom Prospective Diabetes Study [10]. Metformin is now recommended as the first-line treatment for patients with T2DM because abundant studies have shown that it has various beneficial effects beyond glucose-lowering, such as anti-cancer, anti-aging, anti-inflammation and even antibiotic [9].

We recently found that patients with T2DM who had used metformin for more than two years had a significantly lower risk of malignant brain tumors [11]. However, epidemiological data evaluating the effect of metformin on the protection against BBT are still rare. A recent matched case-control study using the UK Clinical Practice Research Datalink showed a higher but not significant risk of meningioma associated with metformin use, with an estimated odds ratio of 1.64 (95% confidence interval: 0.89–3.04) [3]. Because millions of diabetes patients are being treated with metformin, it is clinically important to clarify whether metformin can really increase the risk of BBT. The present study aimed at clarifying the effect of metformin on BBT in patients with T2DM.

## 2. Materials and Methods

The Taiwan government implemented a universal and unique health care system, which has been called the National Health Insurance (NHI) since 1 March 1995. The NHI is characterized by a high coverage rate of 99.6% of Taiwan’s population and a high rate of involvement of medical providers (93% of all medical settings and all in-hospitals). The data that have to be submitted for reimbursement purpose include disease diagnoses, prescribed medications and performed procedures. Researchers can submit proposals for ethics review by using the database for academic research. The present study was approved and supported by the National Science Council (NSC 102-2314-B-002-067) and was reviewed by the National Health Research Institutes with an approval number of NHIRD-102-175 to provide the related database. The database has been described in more detail previously [12,13].

During the whole study period, the International Classification of Diseases, Ninth Revision, Clinical Modification (ICD-9-CM) was the coding system for disease diagnoses in the database. Accordingly, the ICD-9-CM codes for diabetes mellitus were 250.XX and the codes for BBT were 225.X (benign neoplasm of brain and other parts of nervous system). The code 225.2 (benign neoplasm of cerebral meninges) was also used to identify a more specific outcome of cerebral meningioma among the diagnoses of BBT.

**Figure 1:**
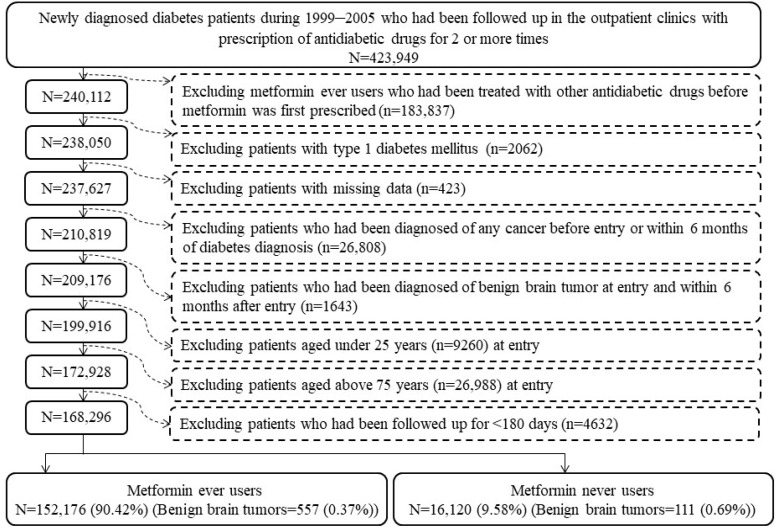
The step-by-step procedures followed in the creation of a cohort of ever users and never users of metformin derived from the Taiwan’s National Health Insurance.

The step-by-step procedures in Figure 1 were used to create a cohort of patients with T2DM enrolled in the study. First, we identified 423,949 patients who were newly diagnosed with diabetes mellitus between 1999 and 2005 and had received two or more times a prescription of antidiabetic drugs in the outpatient clinics. Ever users of metformin were defined as patients who had received metformin as the first antidiabetic drug. Therefore, 183,837 patients who had received a prescription of any other antidiabetic drug before metformin was initiated were first excluded. Ineligible patients who fitted the following criteria were then excluded: (1) 2062 patients who had been diagnosed as having type 1 diabetes mellitus, (2) 423 patients who had missing data, (3) 26,808 patients who had been diagnosed with any cancer before the entry date or within a short period of six months after diabetes diagnosis, (4) 1643 patients who had a previous diagnosis of BBT before enrollment or within 6 months of diabetes diagnosis, (5) 9260 patients aged <25 years, (6) 26,988 patients aged >75 years and (7) 4632 patients who were followed up for a duration of <180 days. As a result, we identified 152,176 ever users and 16,120 never users of metformin for the study.

The prescription information of metformin in the longitudinal database was used to calculate cumulative duration of metformin therapy, expressed in months, for each patient. A potential dose-response effect was evaluated by analyzing the risk in patients categorized according to the tertiles of cumulative duration of metformin therapy.

Potential confounders were classified into demographic and basic data, diabetes-related complications, major comorbidities, antidiabetic drugs and drugs commonly prescribed to patients with diabetes mellitus.

The demographic and basic data included variables of age, sex, living region and occupation. The living region and occupation were detailed elsewhere [14]. In brief, the living region was classified as Taipei, Northern, Central, Southern, and Kao-Ping/Eastern. Occupation was classified as class I (civil servants, teachers, employees of governmental or private businesses, professionals and technicians), class II (people without a specific employer, self-employed people or seamen), class III (farmers or fishermen) and class IV (low-income families supported by social welfare, or veterans).

Diabetes-related complications included nephropathy, eye disease, stroke, ischemic heart disease and peripheral arterial disease. Major comorbidities included hypertension, dyslipidemia, obesity, chronic obstructive pulmonary disease, tobacco abuse, alcohol-related diagnoses and ocular pterygium (used as a surrogate of UV sunlight exposure). The ICD-9-CM codes for the above diagnoses have been described previously [11].

Antidiabetic drugs were classified as insulin, sulfonylurea, meglitinide, acarbose, rosiglitazone and pioglitazone. Drugs commonly prescribed to patients with diabetes mellitus were angiotensin-converting enzyme inhibitor/angiotensin receptor blocker, calcium channel blocker, statin, fibrate and aspirin.

The standardized difference was calculated for each of the potential confounders according to Austin and Stuart [15]. A value of >10% was considered as a threshold for the indication of a potential confounding effect from the variable.

Statistical analyses were conducted for outcomes of any BBT and for cerebral meningioma, respectively. The incidence density of BBT/cerebral meningioma was calculated for the following subgroups of metformin exposure: never users, ever users and ever users classified according to the first, second and third tertile of cumulative duration of metformin therapy. The case number of new-onset BBT/cerebral meningioma diagnosed during the follow-up duration was the numerator of the incidence density. The denominator of the incidence density was the time of follow-up expressed as per 100,000 person-years. Follow-up started on 1 January 2006 and ended on the date of any of the following events whichever occurred first: a new-onset BBT/cerebral meningioma, death or the last reimbursement record, until 31 December 2011. We ended the follow-up by 2011 because the Bureau of NHI started to introduce and promote the use of ICD-10-CM to contracted hospitals and medical settings since 2012. This might have caused a mixture of the use of two disease coding systems.

Propensity score was created by logistic regression by treating all the variables listed in Table 1 and the entry date as independent variables. To reduce confounding by indication, hazard ratios and their 95% confidence intervals were estimated through Cox regression incorporated with the inverse probability of treatment weighting (IPTW) using the propensity score, as proposed by Austin [16].

The following three sensitivity analyses were conducted to examine the consistency of the findings: (1) patients receiving any two consecutive prescriptions of metformin spanning a period of more than four months were excluded. (2) patients having been treated with incretins during follow-up were excluded; and (3) patients having been treated with insulin were excluded. Because the Bureau of the NHI does not allow a prescription of medications for more than 3 months in each outpatient visit, the first sensitivity analyses excluded most patients without receiving regular drug refills. Because incretin-based therapies had not been introduced into Taiwan until after the enrollment of the patients, the second sensitivity analyses were aimed at avoiding the potential influence of these therapies that could have happened after the enrollment of the patients. Patients having been treated with insulin were excluded in the third sensitivity analyses because never users of metformin were characterized by a higher proportion of insulin use (8.34% versus 2.33%, Table 1) and insulin is a growth factor for cell proliferation.

We used SAS statistical software (version 9.4, SAS Institute, Cary, NC, USA) to analyze the data. A *p*-value < 0.05 was considered statistically significant.

## 3. Results

The characteristics between never users and ever users of metformin are compared in Table 1. The following variables had values of standardized difference >10%: age, nephropathy, eye diseases, dyslipidemia, insulin, meglitinide, acarbose, rosiglitazone, pioglitazone, statin and fibrate, suggesting potential risk of confounding from these variables.

Table 2 shows the incidence rates of BBT and cerebral meningioma, respectively, in different subgroups of metformin exposure; and the hazard ratios comparing the exposed subgroups to never users. While comparing metformin ever users to never users, a significantly (50%) lower risk was observed for both BBT and cerebral meningioma. The findings in the tertile analyses were also very similar for BBT and cerebral meningioma, showing a lower risk associated with metformin use in a dose-response pattern. A significant risk reduction could be seen only after 2 years of metformin use as shown in the second and third tertiles of cumulative duration of metformin therapy.

Sensitivity analyses are shown in Table 3. The results are very similar to those of the main analyses in Table 2.

## 4. Discussion

The findings of the present study suggested that metformin use was associated with a significantly lower risk of BBT and cerebral meningioma in patients with T2DM (Table 2 and Table 3). The risk reduction showed a dose-response pattern and was significant after a cumulative duration of metformin therapy of two or more years (Table 2 and Table 3).

The mechanisms of the potential protective effect of metformin on BBT and/or cerebral meningioma remain unknown and await further investigation. Some biological effects of metformin may explain such a protective effect. Metformin may alter the gut microbiota leading to an increased production of butyrate, which may in turn reduce insulin resistance and obesity [9,17,18], the important risk factors of meningioma. Meningiomas are characterized by activation of multiple growth factor signaling pathways involving excess expression of membrane receptors of insulin-like growth factor receptor, platelet-derived growth factor receptor and vascular endothelial growth factor receptor [19]. Metformin improves insulin effect and may reverse the proliferative effects of these growth factors, via AMPK activation and inhibition of the mammalian target of rapamycin pathway [20,21,22,23,24].

There are some clinical implications in the present study. First, together with the finding of a protective effect of metformin on malignant brain tumors seen in our previous study [11], metformin might also provide a protective effect on the development of BBT, in terms of cerebral meningioma and/or other types of BBT (Table 2 and Table 3). These observations suggested that there might be some common pathophysiological pathways involved in the development of either malignant or benign brain tumors. This extra benefit of metformin further strengthened the recommendation of metformin as the first-line therapy for T2DM. Second, the significant risk reduction observed after two years of metformin use and the dose-response pattern provided good reasons to consider the continuous use of metformin when additional antidiabetic drugs are required to more adequately lower blood glucose levels during the course of treatment. Third, several clinical trials are being conducted to evaluate the efficacy of metformin on the treatment of malignant brain tumors [25,26,27]. However, the usefulness of metformin on the prevention and treatment of BBT has not gained similar attention. Although BBT causes less severe clinical problems and is less life-threatening, the findings of the present study provide good rationale for designing and conducting clinical trials to investigate metformin’s efficacy as a preventive agent for BBT and probably also other benign diseases characterized by cellular proliferation.

The present study has carefully addressed the potential methodological limitations commonly seen in pharmacoepidemiological studies that use big databases. These limitations may include selection bias, prevalent user bias, immortal time bias and confounding by indication.

It is believed that the problem of selection bias could be avoided in the present study because the healthcare system of the NHI covers nearly the whole population. Prevalent user bias can result from the enrollment of prevalent users rather than new users of a medication under investigation [28]. Two types of bias can be introduced. First, “prevalent users are ‘survivors’ (healthy users) of the early period of pharmacotherapy, which can introduce substantial selection bias if the risk varies with time.” Second, “covariates for drug use at study entry are often influenced by the previous intake of the drug”. Therefore, prevalent user bias might have existed in the earlier study conducted in the UK that used a matched case-control study design [3]. To mitigate such a problem, a “new user design” is recommended. In the present study, we carefully addressed this problem by enrolling patients at the time of diabetes diagnosis and only new users of metformin were defined in the user group. Additionally, to exclude the potential carry-over effect of other antidiabetic drugs, we enrolled only ever users of metformin who had never been previously treated with other antidiabetic drugs when the patients were first prescribed metformin (Figure 1).

The follow-up period when the outcome cannot happen is considered as the immortal time. When the treatment status is not appropriately assigned or when the follow-up time is not appropriately calculated, immortal time bias can be introduced. By enrolling patients who had two or more times a documented prescription of antidiabetic drugs from the nationwide NHI database (Figure 1), inappropriate assignment of treatment status is not likely. The follow-up time could be simply and probably accurately calculated from the database. The following periods of “immortal time” were not calculated in the follow-up time of the patients: (1) during the initial period of follow-up for <180 days; (2) between diabetes diagnosis and the start of the use of antidiabetic drugs; and (3) follow-up period of patients without use of any antidiabetic drugs (Figure 1). Another potential source of immortal time is the waiting period between the prescription and the dispense of drugs that may happen when a patient is discharged from an admission. Although this may be commonly seen in many other countries, this does not happen in Taiwan’s NHI healthcare system because when the patient is discharged from the hospital, he/she can obtain all discharge medications immediately from the hospital.

Confounding by indication may happen when a risk factor of the outcome is associated with the indication of a medication under investigation [28]. This could be reduced in the study by modeling with Cox regression incorporated with IPTW using propensity score [16].

The consistency of a beneficial effect of metformin on the prevention of BBT/cerebral meningioma and the dose-response pattern in different models (Table 2 and Table 3) strengthened the robustness of the findings of the study.

There are several other strengths in the study. The exclusion of patients with a diagnosis of BBT within 6 months of diabetes diagnosis minimized reverse causality. With the use of existing medical records, self-reporting bias could be avoided. Because the drug cost-sharing is low in our NHI healthcare system, it is believed that detection bias as a result of discrepant socioeconomic status should be minimal. Furthermore, most of the healthcare expenses can be waived when the patients have low incomes, are veterans and receive prescription refills for chronic diseases.

Finally, we recognized that unmeasured confounders could never be adjusted for by statistical methods. Therefore, it is not known whether the results of the study could be biased by a lack of measurement data such as biochemistry, levels of insulin and some growth factors, immune profiles, hormone use, cell phone use, education levels, household conditions, nutritional status, dietary pattern, anthropometric factors, occupational exposure, physical activity, lifestyle, smoking, alcohol drinking and family history. However, a confounder needs to be correlated with the exposure (i.e., metformin use in the present study) and the disease (i.e., BBT/cerebral meningioma in the present study) [29]. Furthermore, it must not be a factor in the causal pathway in-between the exposure and the disease [29]. Although the unmeasured variables may be risk factors of BBT/cerebral meningioma (disease), there is no evidence to support that they fit the other criteria to exert a confounding effect. Furthermore, the lack of histopathological data is another potential limitation associated with the study. It is worth pointing out that knowledge of absolute risk reduction and number needed to treat is important for decision making and clinical application [30]. As the incidence of BBT was low, the absolute risk reduction calculated was too small (111/16,120 − 557/152,176 = 0.32%) and the number needed to treat (the reciprocal of absolute risk reduction) of 310 seemed to be too large as to be cost-effective to use metformin for the prevention of BBT, especially in people without diabetes mellitus.

In conclusion, this study supports a lower risk of BBT/cerebral meningioma in patients with T2DM who have used metformin, especially when metformin has been used for more than 2 years. However, additional studies are required to confirm the findings with more appropriate consideration of measured confounders and histopathological types. Because metformin is cheap and has a very safe profile with no risk of hypoglycemia, the usefulness of metformin in the prevention or treatment of BBT is worthy of in-depth investigation, in either diabetes patients or people without diabetes.

## Figures and Tables

**Table 1:** Comparisons of characteristics between never users and ever users of metformin.

Variables	Never Users		Ever Users		Standardized Difference
	*n* %		*n* %		
**Demographic and basic data**					
Age * (years)	63.62	10.42	61.82	10.03	−17.94
Sex (men)	9251	57.39	82,023	53.90	−7.72
Occupation					
I	6292	39.03	59,414	39.04	
II	3206	19.89	34,993	23.00	8.11
III	3393	21.05	31,893	20.96	−0.04
IV	3229	20.03	25,876	17.00	−8.68
Living region					
Taipei	5411	33.57	48,057	31.58	
Northern	1647	10.22	17,233	11.32	3.72
Central	2817	17.48	27,845	18.30	2.24
Southern	2804	17.39	25,854	16.99	−1.12
Kao-Ping and Eastern	3441	21.35	33,187	21.81	1.33
**Diabetes-related complications**					
Nephropathy	5618	34.85	41,991	27.59	−17.82
Eye diseases	2989	18.54	49,367	32.44	32.45
Stroke	5323	33.02	45,137	29.66	−8.06
Ischemic heart disease	7700	47.77	70,025	46.02	−3.72
Peripheral arterial disease	3744	23.23	3744	2.46	6.62
**Major comorbidities**					
Hypertension	13,211	81.95	124,832	82.03	0.30
Dyslipidemia	11,642	72.22	126,294	82.99	28.48
Obesity	437	2.71	6875	4.52	9.93
Chronic obstructive pulmonary disease	8029	49.81	74,215	48.77	−2.52
Tobacco abuse	454	2.82	6100	4.01	6.80
Alcohol-related diagnoses	1280	7.94	10,862	7.14	−4.40
Ocular pterygium	892	5.53	8901	5.85	1.37
**Antidiabetic drugs**					
Insulin	1344	8.34	3545	2.33	−30.64
Sulfonylurea	11,739	72.82	110,722	72.76	5.43
Meglitinide	1322	8.20	5985	3.93	−19.09
Acarbose	1810	11.23	8335	5.48	−20.41
Rosiglitazone	476	2.95	7549	4.96	10.87
Pioglitazone	395	2.45	4031	2.65	−20.41
**Drugs commonly prescribed to patients with diabetes mellitus**					
Angiotensin converting enzyme inhibitor/angiotensin receptor blocker	11,216	69.58	111,734	73.42	8.91
Calcium channel blocker	10,129	62.83	91,610	60.20	−5.57
Statin	8710	54.03	100,459	66.02	26.35
Fibrate	5512	34.19	65,889	43.30	19.99
Aspirin	9248	57.37	94,075	61.82	9.44

* Age is expressed as mean and standard deviation. The classifications of occupation are described in “Materials and Methods”.

**Table 2:** Incidence rates of benign brain tumors by metformin exposure and hazard ratios comparing exposed to unexposed subgroups.

Metformin Use	Incident Case Number	Cases Followed	Person-Years	Incidence Rate (per 100,000 Person-Years)	Hazard Ratio	95% Confidence Interval	*p* Value
**All benign brain tumors**							
Never users	111	16,120	72,101.43	153.95	1.000		
Ever users	557	152,176	717,670.51	77.61	0.502	(0.409–0.615)	<0.0001
Tertiles of cumulative duration of metformin therapy (months)							
Never users	111	16,120	72,101.43	153.95	1.000		
<27.10	260	50,218	176,390.46	147.40	0.910	(0.728–1.138)	0.4096
27.10–58.27	182	50,219	245,729.50	74.07	0.475	(0.375–0.602)	<0.0001
>58.27	115	51,739	295,550.54	38.91	0.243	(0.187–0.315)	<0.0001
**Cerebral meningioma**							
Never users	21	16,120	72,382.18	29.01	1.000		
Ever users	106	152,176	718,771.52	14.75	0.506	(0.317–0.808)	0.0044
Tertiles of cumulative duration of metformin therapy (months)							
Never users	21	16,120	72,382.18	29.01	1.000		
<27.10	46	50,218	176,960.29	25.99	0.895	(0.531–1.508)	0.6771
27.10–58.27	42	50,219	246,090.39	17.07	0.585	(0.346–0.988)	0.0451
>58.27	18	51,739	295,720.85	6.09	0.196	(0.104–0.369)	<0.0001

**Table 3:** Sensitivity analyses.

Metformin Use	Incident Case Number	Cases Followed	Hazard Ratio	95% Confidence Interval	*p* Value
**All benign brain tumors** Patients who had not received regular refill of metformin * were excluded					
Never users	111	16,120	1.000		
Ever users	140	51,209	0.402	(0.313–0.515)	<0.0001
Tertiles of cumulative duration of metformin therapy (months)					
Never users	111	16,120	1.000		
<27.10	49	16,728	0.615	(0.437–0.865)	0.0052
27.10–58.27	45	13,916	0.453	(0.320–0.640)	<0.0001
>58.27	46	20,565	0.253	(0.179–0.358)	<0.0001
Patients treated with incretins after start of follow-up ** were excluded					
Never users	110	15,148	1.000		
Ever users	520	116,300	0.593	(0.483–0.729)	<0.0001
Tertiles of cumulative duration of metformin therapy (months)					
Never users	110	15,148	1.000		
<27.10	248	42,321	0.980	(0.782–1.229)	0.8638
27.10–58.27	171	38,037	0.562	(0.442–0.714)	<0.0001
>58.27	101	35,942	0.295	(0.225–0.387)	<0.0001
**Patients treated with insulin were excluded**					
Never users	106	14,776	1.000		
Ever users	540	148,631	0.483	(0.392–0.594)	<0.0001
Tertiles of cumulative duration of metformin therapy (months)					
Never users	106	14,776	1.000		
<27.10	257	48,735	0.896	(0.713–1.125)	0.3438
27.10–58.27	172	49,101	0.446	(0.350–0.568)	<0.0001
>58.27	111	50,795	0.231	(0.177–0.302)	<0.0001
**Cerebral meningioma** Patients who had not received regular refill of metformin * were excluded					
Never users	21	16,120	1.000		
Ever users	19	51,209	0.287	(0.155–0.535)	<0.0001
Tertiles of cumulative duration of metformin therapy (months)					
Never users	21	16,120	1.000		
<27.10	8	16,728	0.572	(0.250–1.309)	0.1862
27.10–58.27	8	13,916	0.429	(0.189–0.969)	0.0419
>58.27	3	20,565	0.089	(0.026–0.298)	<0.0001
Patients treated with incretins after start of follow-up ** were excluded					
Never users	21	15,148	1.000		
Ever users	94	116,300	0.565	(0.352–0.906)	0.0179
Tertiles of cumulative duration of metformin therapy (months)					
Never users	21	15,148	1.000		
<27.10	43	42,321	0.941	(0.555–1.594)	0.8209
27.10–58.27	37	38,037	0.645	(0.377–1.103)	0.1090
>58.27	14	35,942	0.211	(0.107–0.416)	<0.0001
**Patients treated with insulin were excluded**					
Never users	21	14,776	1.000		
Ever users	102	148,631	0.462	(0.289–0.739)	0.0013
Tertiles of cumulative duration of metformin therapy (months)					
Never users	21	14,776	1.000		
<27.10	46	48,735	0.852	(0.506–1.435)	0.5473
27.10–58.27	39	49,101	0.516	(0.303–0.877)	0.0146
>58.27	17	50,795	0.175	(0.092–0.333)	<0.0001

* Defined as “patients who had received two consecutive metformin prescriptions spanning a period of more than four months.” ** Incretin-based therapies were not available in Taiwan before the starting date of follow-up.

## Data Availability

The datasets presented in this article are not readily available because public availability of the dataset is restricted by local regulations to protect privacy.
